# Acaricidal Properties of Four Neem Seed Extracts (*Azadirachta indica*) on the Camel Tick *Hyalomma dromedarii* (*Acari: Ixodidae*)

**DOI:** 10.3389/fvets.2022.946702

**Published:** 2022-07-22

**Authors:** Ahmed Gareh, Dalia Hassan, Asmaa Essa, Saber Kotb, Mohammed Karmi, Abou El-Hamd H. Mohamed, Abeer Mousa Alkhaibari, Elzahara Elbaz, Nagwa M. Elhawary, Eman A. A. Hassanen, Maha S. Lokman, Fatma A. El-Gohary, Ehab Kotb Elmahallawy

**Affiliations:** ^1^Department of Parasitology, Faculty of Veterinary Medicine, Aswan University, Aswan, Egypt; ^2^Department of Animal and Poultry Hygiene, Faculty of Veterinary Medicine, Assiut University, Assiut, Egypt; ^3^Department of Animal and Poultry Hygiene, Faculty of Veterinary Medicine, Aswan University, Aswan, Egypt; ^4^Department of Food Hygiene, Faculty of Veterinary Medicine, Aswan University, Aswan, Egypt; ^5^Chemistry Department, Faculty of Science, Aswan University, Aswan, Egypt; ^6^Department of Biology, Faculty of Science, University of Tabuk, Tabuk, Saudi Arabia; ^7^Department of Internal Medicine and Infectious Diseases, Faculty of Veterinary Medicine, Mansoura University, Mansoura, Egypt; ^8^Department of Parasitology, Faculty of Veterinary Medicine, Kafrelsheikh University, Kafr El Sheikh, Egypt; ^9^Department of Parasitology, Faculty of Veterinary Medicine, Zagazig University, Zagazig, Egypt; ^10^Department of Biology, College of Science and Humanities in Al-Kharj, Prince Sattam Bin Abdulaziz University, Al-Kharj, Saudi Arabia; ^11^Department of Zoology and Entomology, Faculty of Science, Helwan University, Cairo, Egypt; ^12^Department of Hygiene and Zoonoses, Faculty of Veterinary Medicine, Mansoura University, Mansoura, Egypt; ^13^Department of Zoonoses, Faculty of Veterinary Medicine, Sohag University, Sohag, Egypt

**Keywords:** acaricidal properties, neem seed extracts, eggs, immature, adult, camel tick

## Abstract

Tick infestation remains one of the major health problems that affect the productivity and comfort of camels. The control of ticks mainly relies on using chemical acaracides. Limited information is available on the potential benefits and activity of various neem extracts on *Hyalomma* ticks. The present study investigated the acaricidal activity of neem seed extracts at different concentrations against developmental stages of the camel tick *Hyalomma dromedarii* in comparison to Butox and diazinon. The acaricidal activity of three extracts, namely, hexane extract (HE), methyl chloride extract (MCE), and methanol extract (ME), of neem seeds (*Azadirachta indica*) were tested at varying concentrations of 5, 10, 15, and 20% on engorged *H. dromedarii* female ticks at days 1, 3, 5, 7, 12, 16, 20, 28, 37, and 43 after treatment (DPT). Interestingly, results of applying different neem seed extracts to engorged *H. dromedarii* female ticks showed that the most effective extract was hexane at concentration 20%, causing 100% mortality at 1st day post-application, while methanol extract at 20% and dichloromethane extract at 20% caused the death of all ticks at 28th day posttreatment as compared to Butox^®^ 5.0 and Diazinon-60, which resulted in mortality of all ticks at 3 and 5 DPT, respectively. In addition, no mortality was reported with the application of aqueous extract (AE), which served as the control group. Furthermore, the neem hexane extract exhibited high efficacy against reproductive performance of female ticks, whereas no fertility or oviposition was reported at all of their concentrations. Additionally, no hatchability occurred using all neem extracts, except the aqueous extract, which showing no effect. In the present study, larvae responded more rapidly to the plant extracts, whereas mortality of all larvae was recorded at 24 h after treatment with 5% hexane. Taken together, this study pointed out that the acaricidal effect of hexane extract of neem seeds was more effective and could be economically used for controlling *H. dromedarii* ticks.

## Introduction

Camels are considered elemental part in the sophistication and farming of many countries around the world, being a good source of milk and meat, and serve as a means of transportation (1, 2). However, their productivity is hindered by a wide range of external and internal parasites, resulting in considerable economic losses (1, 3–6). Ticks are destructive blood-sucking ectoparasites of worldwide distribution and of a greater economic importance in tropical and subtropical areas (7, 8). Ticks have been considered potent vectors for the transmission of various bacterial, viral, and parasitic diseases of veterinary and medical importance. Therefore, ticks are considered a major contributor for several emerging and re-emerging diseases (9, 10). Given its important economic impacts, tick infestation in camels might result in a series of symptoms, ranging from mild to severe anemia; intense pruritus and deterioration the skin of affected animals; loss of appetite, leading to a reduction in growth rate and decreased productivity; and occasional mortalities in untreated and young animals, and therefore, they result in considerable economic losses (11, 12). To the authors' knowledge, *Hyalomma dromedarii* is considered the most reported *Hyalomm*a spp. parasitizing camels in Egypt (13). The camel is considered the primary host containing the adult stage of *Hyalomm*a spp.; however, it also infests other domestic animals, including cattle, sheep, goats, and equids. The remaining stages, including larvae and nymphs, feed on birds and small burrowing animals, but nymphs can infest large animals such as adults and complete its life cycle on two or three hosts (14).

The control of ticks is mainly based on the direct application or injecting of acaricides to animals. Several acaricides have been extensively used and recommended for the control of ticks including organophosphates, carbamates, pyrethroids, and amidines (15). However, several major drawbacks were reported for the majority of acaricides including alarming reports of tick resistance, residues in foods, and environmental pollution (16, 17). It is therefore not surprising to mention that there is an urgent need to develop eco-friendly effective alternatives for these chemicals. Interestingly, the use of herbal medications becomes a promising alternative approach for the treatment of various infectious agents because of their biodegradability, target efficiency, and cost-effectiveness, and therefore, they gained a considerable interest in tropical and subtropical regions (18–26). Among others, plant-derived materials and their bioactive substances were proposed as substitutes for synthetic acaricides due to their activity against ticks (27). As compared to synthetic ones, several previous reports revealed that herbal acaricides caused little environmental pollution and a low toxicity level to non-target organisms including humans, apart from the rapid biodegradation of their residues and their role in prevention of resistance development (28, 29).

Neem is considered one of the most reliable botanical sources of biopesticides with a wide range of biological activities (30). The neem tree, *Azadirachta indica A. Juss*, is an evergreen tree that originates from India and other neighboring countries (31–33). The functional ingredients of neem, including neem oil, bark, leaves, and their purified biochemical products, exhibited promising therapeutic effects and showed anticancer, anti-inflammatory, and antimicrobial properties (32–35). It is noteworthy to state that neem and its extracts showed potent activity against all the stages (adult, nymph, and larva) of ticks (29, 36, 37). Azadirachtin analogs, including azadirachtin A and its B counterpart, are among the most potent constituents isolated from the crude extracts of neem (38, 39). These analogs exhibited a wide range of biological properties, which include antifeedant, larvicidal, ovicidal, repellent action, growth deregulation, reduction in ecdysone levels, alterations in development and reproduction, sterility, and damage in molting processes (40). In accordance with its mechanisms of action against insects, neem is structurally analogous to insect hormones known as “ecdysone,” which are responsible for metamorphosis in insects. It is therefore not surprising to mention that the larvicidal and acaricidal properties of neem are the underlying bases for the use of neem products for control of agricultural pests.

Revising the available literature, limited information is available about the use of *A. indica* (neem) extracts against *H. dromedarii*. A previous study (41) showed the *in vitro* acaricidal activity of the methanolic extract of neem leaves against engorged adult female ticks, egg hatchability, and larvae of camel ticks (*H. dromedarii*) using the immersion method and in mortality rates of engorged ticks from 1st to 15th DPT (up to 100%), with some changes in morphology. Also, there was a potent activity of the methanolic extract of neem leaves on hatchability of *H. dromedarii* eggs (100%) from 1st to 15th DPT, and it induced 100% mortality on the newly hatched larvae of *H. dromedarii* ticks. Agreed with the aforementioned information, this study aimed to investigate the effect of neem extract application on ticks infesting one-humped camels in Aswan Governorate, Egypt, which might provide new insights into the control of hard ticks in camels.

## Materials and Methods

### Ethical Considerations

The ethical approval of the present study was obtained from the Research, Publication, and Ethics Committee of the Faculty of Veterinary Medicine, Aswan University, Egypt, and the Institutional Review Approval Board Number is 2020/5.

### Collection of Hard Ticks

Engorged adult, larvae, and nymphs of hard ticks were collected from naturally infested camels (5- to 15-year-olds) from Daraw market, Daraw city, Aswan Governorate, Egypt. In order to minimize damage to the mouthparts and cuticle, the ticks were manipulated by rotating for easy removal with a pair of soft forceps. The collected ticks were then placed in a clean plastic container with perforated lids to allow ventilation, then immediately transported to the laboratory at the Department of Parasitology, Faculty of Veterinary Medicine, Aswan University, for identification using standardized international keys and bioassays, as described elsewhere (14, 42, 43).

### Chemical Materials

Hexane, diethyl ether, dichloromethane, and methanol solvents, Tween 20, Tween 80, TLC plates 20 × 20 cm, and vanillin spray reagent were purchased from Sigma Chemical Company, Cairo, Egypt. Synthetic chemical acaricides such as deltamethrin (Butox 5%) and Diazinon-60 were purchased from Arab Chemical Industrial Company, Cairo, Egypt.

### Plant Preparation and Extraction

#### Collection of Seeds

Neem seeds were collected from an old neem tree located at Aswan University during July 2020 at 10 a.m. and identified and authenticated by Department of Botany, College of Science, Aswan University. The collected seeds were then transported to the Laboratory of Parasitology, Faculty of Veterinary Medicine, Aswan University, Egypt, and stored at 4°C until analysis.

#### Preparation of Seeds

The seeds were cleaned to remove any sticks, unwanted leaves, bad seeds, sand and dirt in order to ensure the oil produced is not contaminated and of high quality. The cleaned neem seeds were dried at 55°C for 72 h until constant weight and moisture content determined, as described elsewhere (44) using Equation 1:


$$\begin{array}{l} \text{Moisture content}=\frac{\text{W}1\text{ W}2}{\text{W}1}\;\times 100\% \end{array}$$

where W1 is the weight of neem seeds before drying and W2 is the weight of neem seeds after drying.

The dried clean neem seeds were roasted about 5 min to enhance extraction of oil, then crushed in a blender, and sieved to obtain particles ranging from 425 to 710 μm in size. The sieved neem powder was then stored under vacuum in an airtight container at 4°C prior to use (45).

#### Neem Seed Extraction by Cold Maceration

To obtain extracts from the neem seeds, about 9,000 ml of hexane was added to 3,000 g of the dried grounded neem seeds in a conical flask, which is then allowed to stand at room temperature for a period of 3 days with intermittent agitation (stirring) until the soluble matter dissolved. This mixture was filtered by gravity filtration, producing a hexane extract mixture as the filtrate, which was then concentrated using a rotary evaporator and stored at 4°C until further use (46). For the study, three more consecutive extracts, including dichloromethane, methanol, and aqueous extracts from residues have been collected using the same method. The yielded neem oil was calculated elsewhere (47) using the following equation (Equation 2).


$$\begin{array}{l} \text{Extraction yield }\left(\%\right)\;=\frac{\text{M}1-\text{M}2}{\text{M}1}\;\times 100\% \end{array}$$

where M1 is the mass of the neem seed before extraction and M2 is the mass of the neem seed after extraction.

#### Preparation of Emulsions

The emulsion was prepared by mixing neem oil and two different non-ionic surfactants (Tween 20 and Tween 80) using each emulsifier separately, at rates of 1:5 and 1:3, respectively. Surfactants and deionized water were first mixed using a stirrer, and then neem oil was added. This step was followed by preparation of the different concentrations (5, 10, 15, and 20%) from each extracts as described elsewhere (48) with slight modification in which Tween 20 and Tween 80 were replaced by soap as a surfactant for complete blending of neem oil with water.

#### Preparation of Thin-Layer Chromatography (TLC) Plates

In this step, the plant extract was spotted on the plate with the aid of a capillary tube. The spot was applied 1 cm upward from the lower end of the TLC plate, then placed in a beaker consisting hexane:ether [(1:1), (2:1), (3:1), (5:1), and (6:1)] with drops of methanol and dichloromethane:methanol (7:1/4) as the mobile phase. Run spots were performed to separate the compounds. Later on, when the mobile phase reached the upper end of the TLC plate, it was removed from a beaker and was air dried. The TLC run plates were observed by using a UV spectrophotometer, and the separated spots were marked. Then, the spots were visualized by vanillin spray reagent. After spraying, the plates were immediately placed in the oven maintained at 1,100°C for 5–10 min (49). A preparative thin-layer chromatographic separation of the dichloromethane extract was applied.

### *In vitro* Evaluation the Acaricidal Effect of Neem on Hard Ticks of Camels

#### *In vitro* Evaluation of Acaricidal Effect of Neem Seed Extracts on Adult Female *H. dromedarii*

Four concentrations (5, 10, 15, and 20%) of neem extract emulsions were used, while in case of aqueous emulsion, only two concentrations of 5 and 10% were tested. Also, 5% deltamethrin (Butox 5%) and Diazinon-60 were applied. The tested groups included 15 adult female ticks (three replicates for each concentration) that were weighed and immersed in their respective dilutions for 5 min. The adult immersion test was then performed by placing each group of ticks in separate petri dishes, and all plates were incubated at 27–30°C and 70%−80% relative humidity (RH). A negative control composed of the surfactant and distilled deionized water was included along the study. The number of live and dead ticks was counted during the period of 43 days (posttreatment period). The mortality rate of adult female ticks was calculated according to the following equation (Equation 3):


$$\begin{array}{l} \text{Mortality rate}=\frac{\text{Number of dead ticks}}{\text{Total number of ticks}}\times 100 \end{array}$$

The acaricidal efficacy was calculated using the following equation (50), and the index of egg laying was calculated after completed oviposition (36 days), using the following formula (Equation 4), as described elsewhere (51):


$$\begin{array}{l} \text{Index of egg laying }\left(\text{IE}\right)=\frac{\text{Mean weight of eggs laid }\left(\text{g}\right)}{\text{Weight of females }\left(\text{g}\right)} \end{array}$$

The eggs were weighted and then incubated in test tubes at 27–30 °C and 70–80% RH. Later on, the percentage inhibition of egg laying was calculated after hatching 21 days by using the following formula (Equation 5):


$$\begin{array}{l} \text{Inhibition of egg laying }\left(\text{\%}\right)=\frac{\text{IE control group}-\text{IE treated group}}{\text{IE control group}}\times 100. \end{array}$$

#### Evaluation the Acaricidal Effect of Neem Seed Extracts on the Eggs of *H. dromedarii*

The neem extract emulsions with the different concentrations (5, 10, 15, and 20%) were applied to its corresponding group of eggs, which were laid by a control group. The eggs (5-day-old eggs) were immersed in neem seed extract emulsions for 5 min and then were placed in open test tubes until drying emulsions, and then the test tubes were closed with cotton plugs to avoid contamination. All tubes were also incubated at 27–30°C and RH of 70%−80%. The control group eggs consisted of filter paper envelops immersed in the surfactant, and distilled deionized water was also used. After 21 days, all tubes were incubated at 40°C for counting, and hatchability was determined, as described elsewhere (52).

#### Evaluation of Acaricidal Effect of Neem Seed Extracts on the Larva of *H. dromedarii*

The eggs of the adult control group were placed in test tubes and incubated at 27–30°C and 70%−80% RH until they hatched into larvae. The larval packet test was performed using about 300–400 larvae (at 10-day-old larvae) which were then placed in filter paper envelopes immersed in neem seed extract emulsions with the different concentrations (5, 10, 15, and 20%) (53). All envelopes were kept under the same incubating conditions. Reading of the results was recorded under UV light with a magnifying glass, and larvae with no movement were considered dead. Furthermore, the mortality rate was calculated as described elsewhere (54) according to the following equation (Equation 6):


$$\begin{array}{l} \text{Mortality rate}=\frac{\text{Number of dead ticks}}{\text{Total number of ticks}}\times 100 \end{array}$$

#### Evaluation of Acaricidal Effect of Neem Seed Extracts on Nymphs of *H. dromedarii*

This step involved collection of nymphs from camel tail and ears. Each group of 15 nymphs was immersed in the various concentrations (5, 10, 15, and 20%) of neem extract emulsions for 5 min. Then, the nymphs were placed in separate petri dishes, and all plates were incubated at 27–30°C and 70%−80% RH. A negative control consisted of the surfactant, and distilled deionized water was also included. The number of live and dead nymphs of ticks was counted posttreatment during the whole time of the test, and the acaricidal efficacy was determined (50), using the following equation (Equation 7), and this step was performed on three replicates for each concentration.


$$\begin{array}{l} \text{Mortality rate}=\frac{\text{Number of dead ticks}}{\text{Total number of ticks}}\times 100. \end{array}$$

### Statistical Analysis

The data related to effects of treatment using different concentrations of various neem seed extracts against the different stages of ticks (eggs, larva, nymph, and adult) and non-hatching eggs and antifeedant activity of the ticks were analyzed by one-way ANOVA. This was followed by using of Duncan multiple range tests (55) to determine the significant differences between treatments, and the least significant difference (LSD) test was used to separate the mean values, which were significant at the 95% level with Statistical Package for the Social Sciences (SPSS version 25). The level of significant association between treatments was considered at *p* < 0.05. Curves and the lethal concentrations (LC50 and LC90) were obtained at 95% using probit analysis with LDP line software (56).

## Results

### Acaricidal Activity of Neem Seeds at Different Concentrations Against Engorged *Hyalomma dromedarii*

Data presented in Table 1 show the effect of neem seed extracts, hexane extract (HE), dichloromethane extract (DE), methanol extract (ME), and aqueous extract (AE), used at a concentration of 5% as compared to the effect of Butox 5% and Diazinon-60 against *H. dromedarii* infestation in camels. As shown, the 5% hexane extract was the most effective extract, which showed evident changes and caused death of *Hyalomma* ticks from the first day of application with a percentage of 46.7%, and caused complete mortality of ticks at day 20. The methanol extract (ME) followed HE in its efficacy since and caused 100% mortality at day 37. On the other hand, little effect was obtained by uding dichloromethane, compared to that recorded with Butox^®^ 5.0 and Diazinon-60, which caused mortality of all ticks at day 3 and day 5, respectively. By contrast, no mortality was recorded among control groups and following treatment with aqueous extracts. The data presented in Table 2 show that hexane extract 10% was the most effective extract, causing mortality to 90% of hard ticks on the first day of application and the death of all ticks at day 5, followed by methanol extract which resulted in 100% mortality at day 28, while little effect was obtained by dichloromethane extract. On the other hand, no mortality was recorded among control groups and because of treatment with aqueous extracts. Data presented in Table 3 indicate that 15% concentration of hexane extract of neem seeds revealed significantly higher mortality (90%) of ticks at the first day of application and 100% mortality at the third day, followed by 15% methanol extract which caused 100% mortality at day 28, while little effect was obtained by 15% dichloromethane extract. As shown in Table 4, 20% concentration of hexane extract of neem seeds was the most toxic extract and triggered 100% mortality of ticks at the first day of application, while the same concentration of methanol and dichloromethane extracts evoke a similar effect but at day 28.

**Table 1:** *In vitro* mortality rates of engorged *Hyalomma dromedarii* treated with 5% hexane, methanol, dichloromethane, and aqueous extracts of neem seeds in comparison to some synthetic chemical materials (Butox 5%, Diazinon-60).

**Group**	**Days post treatment**									
	**1**	**3**	**5**	**7**	**12**	**16**	**20**	**28**	**37**	**43**
HE	46.7^ef^	53.3^def^	60^de^	66.7^cd^	86.7^ab^	93.3^ab^	100^a^	–	–	–
DE	0^h^	0^h^	6.7^gh^	13.3^gh^	20^g^	20^g^	20^g^	20^g^	66.7^cd^	100^a^
ME	0^h^	0^h^	13.3^gh^	20^g^	40^f^	40^f^	40^f^	80^bc^	100^a^	–
AE	0^h^	0^h^	0^h^	0^h^	0^h^	0^h^	0^h^	0^h^	80^bc^	100^a^
C	0^h^	0^h^	0^h^	0^h^	0^h^	0^h^	0^h^	0^h^	66.7^cd^	100^a^
B	86.7^ab^	100^a^	–	–	–	–	–	–	–	–
D	53.3^def^	80^bc^	100^a^	–	–	–	–	–	–	–

*Small superscript letters (a, b, c, d, e, f, g, and h) in the columns and the rows denote significant difference (P < 0.05)*.

*DPT, days posttreatment; DE, dichloromethane neem seed extract emulsion; HE, hexane neem seed extract emulsion; AE, aqueous neem seed extract emulsion; ME, methanol neem seed extract emulsion; C, control; D, Diazinon; B, Butox*.

**Table 2:** *In vitro* mortality rates of engorged *Hyalomma dromedarii* treated with 10% hexane, methanol, and dichloromethane extracts of neem seeds.

**Group**	**Days post treatment**									
	**1**	**3**	**5**	**7**	**12**	**16**	**20**	**28**	**37**	**43**
HE	90^a^	90^a^	100^a^	–	–	–	–	–	–	–
DE	0^f^	0^f^	10^e^	10^e^	10^e^	10^e^	10^e^	10^e^	100^a^	–
ME	40^d^	40^d^	50^c^	80^b^	90^a^	90^a^	90^a^	100^a^	–	–
AE	0^h^	0^h^	0^h^	0^h^	0^h^	0^h^	0^h^	0^h^	80^bc^	100^a^
C	0^f^	0^f^	0^f^	0^f^	0^f^	0^f^	0^f^	0^f^	66.7^c^	100^a^

*Small superscript letters (a, b, c, d, e, and f) in the columns and the rows denote significant difference (P < 0.05)*.

*HE, hexane neem seed extract emulsion; DE, dichloromethane neem seed extract emulsion; ME, methanol neem seed extract emulsion; C, control; AE, aqueous neem seed extract emulsion*.

**Table 3:** *In vitro* mortality rates of engorged *Hyalomma dromedarii* treated with 15% hexane, methanol, and dichloromethane extracts of neem seeds.

**Group**	**Days post treatment**									
	**1**	**3**	**5**	**7**	**12**	**16**	**20**	**28**	**37**	**43**
HE	90^a^	100^a^	–	–	–	–	–	–	–	–
DE	0^f^	10^e^	10^e^	10^e^	10^e^	10^e^	10^e^	10^e^	100^a^	–
ME	40^d^	40^d^	50^c^	80^b^	90^a^	90^a^	90^a^	100^a^	–	–
AE	0^h^	0^h^	0^h^	0^h^	0^h^	0^h^	0^h^	0^h^	80^bc^	100^a^
C	0^f^	0^f^	0^f^	0^f^	0^f^	0^f^	0^f^	0^f^	66.7^c^	100^a^

*Small superscript letters (a, b, c, d, e, and f) in the columns and the rows denote significant difference (P < 0.05)*.

*HE, hexane neem seed extract emulsion; DE, dichloromethane neem seed extract emulsion; ME, methanol neem seed extract emulsion; C, control; AE, aqueous neem seed extract emulsion*.

**Table 4:** *In vitro* mortality rates of engorged *Hyalomma dromedarii* treated with 20% hexane, methanol, and dichloromethane extracts of neem seeds.

**Group**	**Days post treatment**									
	**1**	**3**	**5**	**7**	**12**	**16**	**20**	**28**	**37**	**43**
HE	100^a^	–	–	–	–	–	–	–	–	–
DE	0^d^	20^c^	20^c^	20^c^	20^c^	20^c^	20^c^	100^a^	–	–
ME	70^b^	80^ab^	90^a^	90^a^	90^a^	90^a^	90^a^	100^a^	–	–
AE	0^h^	0^h^	0^h^	0^h^	0^h^	0^h^	0^h^	0^h^	80^bc^	100^a^
C	0^d^	0^d^	0^d^	0^d^	0^d^	0^d^	0^d^	0^d^	66.7^b^	100^a^

*Small superscript letters (a, b, c, d, e, and f) in the columns and the rows denote significant difference (P < 0.05)*.

*HE, hexane neem seed extract emulsion; DE, dichloromethane neem seed extract emulsion; ME, methanol neem seed extract emulsion; C, control; AE, aqueous neem seed extract emulsion*.

### Effects of Various Concentrations of Neem Seed Extracts on Oviposition, Fertility, and Hatchability

Table 5 shows the reproductive performance, including oviposition and fertility percentage, of *H. dromedarii* engorged female ticks exposed to the effect of various concentrations of neem seed extracts. The results indicated that there was no oviposition with all concentrations of hexane extract, while oviposition percentages with application of methanol extract at concentrations of 5, 10, 15, and 20% were 18.8, 12.5, 7.5, and 2.5%, respectively. Furthermore, a little effect was obtained with the application of dichloromethane as the oviposition percentages were 87.5, 81.3, 75, and 68.8% at concentrations of 5, 10, 15, and 20%. The results also indicated that there was a significant effect for hexane extract on the fertility of *H. dromedarii* at all concentrations. In this regard, the fertility rates were 100, 95, 91.6, and 75%, and their corresponding concentrations of methanol extract concentrations were 5, 10, 15, and 20%, respectively. On the other hand, no effect was obtained upon application of aqueous extracts on oviposition and fertility of ticks. Data presented in Table 5 also show that the hatching rates reached 0% with the application of all extract concentrations, except aqueous extract that has no inhibitory effect.

**Table 5:** Effect of 5, 10, 15, and 20% neem seed extracts on reproductive performance of *Hyalomma dromedarii* including oviposition, fertility, and hatchability.

**Criteria**		**Extracts and their concentration**															
		**5%**				**10%**				**15%**				**20%**			
		**HE**	**DE**	**ME**	**AE**	**HE**	**DE**	**ME**	**AE**	**HE**	**DE**	**ME**	**AE**	**HE**	**DE**	**ME**	**AE**
Oviposition		0^a^	87.5^h^	18.8^d^	100^i^	0^a^	81.3^g^	12.5^c^	100^i^	0^a^	75^f^	7.5^b^	100^i^	0^a^	68.8^e^	2.5^a^	100^i^
Fertility		0^a^	100^e^	100^e^	100^e^	0^a^	91.5^d^	95^de^	100^e^	0^a^	75^c^	91.6^d^	100^e^	0^a^	68.2^b^	75^c^	100^e^
Hatchability	Days	HE	DE	ME	AE	HE	DE	ME	AE	HE	DE	ME	AE	HE	DE	ME	AE
	15	0^a^	0^a^	0^a^	0^a^	0^a^	0^a^	0^a^	0^a^	0^a^	0^a^	0^a^	0^a^	0^a^	0^a^	0^a^	0^a^
	21	0^a^	0^a^	0^a^	100^b^	0^a^	0^a^	0^a^	100^b^	0^a^	0^a^	0^a^	100^b^	0^a^	0^a^	0^a^	100^b^
	28	0^a^	0^a^	0^a^	–	0^a^	0^a^	0^a^	–	0^a^	0^a^	0^a^	–	0^a^	0^a^	0^a^	–

*Small superscript letters (a, b, c, d, e, f, g, and h) in the columns and the rows denote significant difference (P < 0.05)*.

*HE, hexane neem seed extract emulsion; DE, dichloromethane neem seed extract emulsion; ME, methanol neem seed extract emulsion; AE, aqueous neem seed extract emulsion; C, control*.

### Effect of Various Concentrations of Neem Seed Extracts on *Hyalomma dromedarii* Larvae and Nymphs

Data presented in Table 6 show that the hexane extract of neem seeds, at all concentrations, induced high mortality rates of newly hatched larvae at the first day posttreatment, followed by methanol extract which caused death of all hatched larvae using concentrations of 10 and 15% first day posttreatment, and the mortality rate was 80% at 1st DPT and reached 100% at the third day of application of 5% methanol extract, while the lowest effect was attained by dichloromethane extract (concentration 5%) which resulted in 65% and 100% mortality of newly hatched larvae at 3rd and 5th days, respectively. Regarding 10% DE, it resulted in 75 and 100% mortality at 1st and 3rd days posttreatment, respectively, while at the concentration of 15%, death of all hatched larvae at 1st DPT occurred. In accordance with nymphs, as shown in Table 7, there was a significant effect of hexane extract and methanol extract on the mortality of *H. dromedarii* nymphs. In this regard, hexane extract at a concentration of 5% induced a mortality rate of nymphs reaching 80 and 100% at the first day posttreatment and 3rd day posttreatment, respectively. Furthermore, the mortality rate of nymphs reached 100% at concentrations of 10 and 15% of hexane extract at first day posttreatment. In the case of methanol extracts, the mortality of nymphs reached to 40 and 100% at the first day posttreatment and 3rd day posttreatment, respectively, while the mortality reached 100% at concentrations of 10 and 15% using the same extract on the first day posttreatment. On the other hand, the lowest effect was observed with dichloromethane, and no effect gained using the aqueous extract.

**Table 6:** *In vitro* mortality rates of *Hyalomma* spp. larvae treated with 5, 10, and 15% of hexane, methanol, dichloromethane, and aqueous extracts emulsions of neem seeds.

**DPT**	**Extracts and their concentration**											
	**5%**				**10%**				**15%**			
	**HE**	**DE**	**ME**	**AE**	**HE**	**DE**	**ME**	**AE**	**HE**	**DE**	**ME**	**AE**
1	100^a^	0^d^	80^b^	0^d^	100^a^	75^b^	100^a^	0^d^	100^a^	100^a^	100^a^	0^d^
3	**–**	65^c^	100^a^	0^d^	**–**	100^a^	**–**	0^d^	**–**	**–**	**–**	0^d^
5	**–**	100^a^	**–**	0^d^	**–**	**–**	**–**	0^d^	**–**	**–**	**–**	0^d^
7	**–**	**–**	**–**	0^d^	**–**	**–**	**–**	0^d^	**–**	**–**	**–**	0^d^
12	**–**	**–**	**–**	0^d^	**–**	**–**	**–**	0^d^	**–**	**–**	**–**	0^d^
20	**–**	**–**	**–**	100^a^	**–**	**–**	**–**	100^a^	**–**	**–**	**–**	100^a^

*Small superscript letters (a, b, c, and d) in the columns and the rows denote significant difference (P < 0.05)*.

*HE, hexane neem seed extract emulsion; DE, dichloromethane neem seed extract emulsion; ME, methanol neem seed extract emulsion; C, control; AE, aqueous neem seed extract emulsion*.

**Table 7:** *In vitro* mortality rates of *Hyalomma* spp. *nymphs* treated with 5, 10, and 15% of hexane, methanol, dichloromethane, and aqueous extract emulsions of neem seeds.

**DPT**	**Extracts and their concentration**											
	**5%**				**10%**				**15%**			
	**HE**	**DE**	**ME**	**AM**	**HE**	**DE**	**ME**	**AE**	**HE**	**DE**	**ME**	**AE**
1	80^ab^	0^f^	40^c^	0^f^	100^a^	0^f^	100^a^	0^f^	100^a^	20^d^	100^a^	0^f^
3	100^a^	0^f^	100^a^	0^f^	–	20^d^	–	0^f^	–	50^bc^	–	0^f^
5	–	10^e^	–	0^f^	–	20^d^	–	0^f^	–	50^bc^	–	0^f^
7	–	10^e^	–	0^f^	–	40^c^	–	0^f^	–	70^b^	–	0^f^
12	–	50^bc^	–	0^f^	–	40^c^	–	0^f^	–	80^ab^	–	0^f^
16	–	70^b^	–	0^f^	–	70^b^	–	0^f^	–	100^a^	–	0^f^
20	–	100^a^	–	70^b^	–	100^a^	–	70^b^	–	–	–	70^b^
28	–	–	–	100^a^	–	–	–	100^a^	–	–	–	100^a^

*Small superscript letters (a, b, c, d, e, and f) in the columns and the rows denote significant difference (P < 0.05)*.

*HE, hexane neem seed extract emulsion; DE, dichloromethane neem seed extract emulsion; ME, methanol neem seed extract emulsion; C, control; AE, aqueous neem seed extract emulsion*.

## Discussion

Chemical acaricides have been considered the main tick control strategy in domestic animals (57). However, the inappropriate use of acaricides and their wider application resulted in an emerging problem of the development of tick resistance to these acaricides (16). Clearly, there is an urgent need for developing environmentally friendly, safe, effective anti-tick natural products that can interrupt the life cycle and all biological processes of insects and dispersal as a herbal acaricide (58, 59). The present study revealed potent acaricidal activity of different concentrations of neem seed extracts against different stages of *H. dromedarii* collected from camels, and their activity seem to depend on the used concentration. As shown in Tables 1–4, the immersion of different concentrations (5, 10, 15, and 20%) of hexane extract (HE) of neem seeds (neem oil) for 5 min resulted in 100% mortality of adult female ticks at 20, 5, 3, and 1 days posttreatment (DPT), respectively. Meanwhile, Butox^®^ 5.0 and Diazinon-60 induced in mortality of all ticks at 3 and 5 DPT, respectively. Furthermore, application of the aqueous extract (AE) of neem seeds caused the mortality of adult ticks 43 DPT, which also occurred in the control groups. The same effect was also observed in previous studies on *Rhipicephalus microplus* (60, 61). Furthermore, another previous report (62) revealed that azadirachtin causes a significant increase in the mortality rate of unfed adults, which reached to 100% on 15th DPT. A previous study documented the potent activity of neem against eggs, immature and adult stages of *Hyalomma anatolicum excavatum* at concentrations of 1.6, 3.2, 6.4, and 12.8%. In the same study, a significant increase in the hatching rate was observed during the first 7 days posttreatment, followed by incompletely developed and dead larvae, and then after 15 days, neem resulted in hatching failure and induced a significant increase in mortality rates of newly hatched larvae, unfed larvae, and unfed adults (63).

As shown in Tables 5–7, the present study revealed that the application of HE induced hatching failure and a high acaricidal effect on oviposition, egg hatchability larvae, and nymphs. Similarly, Al-Rajhy et al. (64) investigated the effects of neem on *Hyalomma anatolicum* ticks and revealed a high acaricidal effect of azadirachtin at low concentrations against larvae and nymphs. Another previous study concluded that various concentrations (10%−100%) of neem seed oil were able to kill all *Boophilus decoloratus* larvae in cattle after a period of 24–27 h (65). By contrast, the obtained results disagree with those of a previous study (64) which reported that azadirachtin had no effects on egg production with a significant reduction in the feeding activity of larvae and a 60% reduction in molting. Another previous study (62) documented the different effects of commercial neem seed oil (Neem Azal F) on *H. anatolicum excavatum* ticks that included an increased hatching rate and earlier hatching before the larvae were fully viable. As shown in our results (Tables 1–4), the used concentrations (5, 10, 15, and 20%) of the dichloromethane extract (DE) of neem seeds exhibited a low acaricidal effect on engorged adult ticks of *H. dromedarii* from the 5th day of application and continued up to an increase at 43th, 37th, 37^th^, and 28th DPT, respectively, resulting in 100% mortality. The possible explanation is the absence of azadirachtin in DE. Regarding the effect of DE against egg hatchability, it had a highly acaricidal effect on egg hatchability (Table 5) at all concentrations and a highly acaricidal effect on larvae at 15% concentration (Table 6), resulting in 100% mortality the 1^st^ DPT. Similar effects were observed by Choudhury (66). The possible explanation might be attributed to the lethal effect of salannin compound (67). Salannin is one of the active components of neem with insect growth-regulating and antifeedant activity since it increases the larval stage duration and causes delayed molt, leading to decreased pupal weight that results in larval and pupal mortality (68). On the other hand, a low acaricidal effect of DE at 15% concentration was reported in nymphs, from the 1st day of application and continued up to 16th DPT, resulting in 100% mortality (Table 7). These findings are consistent with those of a previous study (62) which pointed out that DE contained a large amount of nimbin and salannin (69); nimbin had no significant effect on insects, but salannin had moderate antifeedant and growth-disrupting properties (67). Moreover, DE impaired oviposition at 5, 10, 15, and 20% concentrations by 87.5, 81.25, 75, and 68.75%. In addition, DE impaired the fertility by 100, 91.5, 75, and 68.25% at 5, 10, 15, and 20% concentrations, respectively.

Regarding the effect of methanol extract (ME) of neem seeds on ticks, which is shown in Tables 2–4, ME, at 10, 15, and 20% concentrations, exhibited a highly acaricidal effect against engorged adult ticks of *H. dromedarii* from the 1st day of application and continued up to 28th DPT, reaching 100% mortality. The present results are in agreement with those of a previous study which revealed the acaricidal effect of ME of neem leaves against *H. dromedarii* ticks. Moreover, all concentrations of ME had a lethal effect on oviposition and fertility, with a high acaricidal effect on egg hatchability without influence on hatching rate (Table 5). Our result is in agreement with that reported in several previous studies (41, 60, 70). Moreover, ME exhibited a high acaricidal effect at 10% concentration on larvae and nymphs from 1st DPT (Tables 6, 7), which is consistent with some previous reports (62). Importantly, the present study showed that the AE of neem seeds at all concentrations (Tables 1, 5–7) had no effect on the adult tick of *H. dromedarii*, egg hatchability, oviposition, larvae, and nymphs. These findings are in harmony with data reported by Tamirat et al. (61). This possible explanation of these findings could be attributed to the hypothesis that several polar compounds, like sugars and proteins, are eliminated in the aqueous extract (71). In the present work, the statistical analysis revealed a significant difference in the efficacy and effects of application of Butox (5%), diazinon, 10% hexane, and control group (*p* < 0.05), as well as between 10% hexane extract and Butox (5%) and diazinon (*p* > 0.05), while there is no significant relationship between Butox 5% and diazinon (*p* > 0.05). The tabulated data concluded that the efficiency of Butox 5% and diazinon was more than that of 10% hexane extract against infested ticks. Despite the fact that the data of our current study indicated that synthetic chemical insecticides were more efficient in controlling ticks than neem extract oil, the application of neem at higher doses on affected animals might offer many advantages for the control of ectoparasites without the risk of toxicity to them (72).

## Conclusion

The study concluded that the *in vitro* application of neem extracts showed high efficacy against camel ticks. More importantly, the hexane extract exhibited a highly acaricidal effect on adult ticks of camels from the first day of application and continued up to 20 days after treatment, resulting in 100% mortality. The present data provide a platform for the development of environment-friendly, non-toxic, non-accumulating medicines against ectoparasites, which could be carried out in a large scale in animal farms.

## Data Availability Statement

The original contributions presented in the study are included in the article/supplementary material, further inquiries can be directed to the corresponding author.

## Ethics Statement

The animal study was reviewed and approved by Ethics Committee of the Faculty of Veterinary Medicine, Aswan University, Egypt, and the Institutional Review approval Board Number is 2020/5.

## Author Contributions

AG, DH, AE, SK, MK, and AM designed the idea of the conception, performed the methodology, formal analysis, data curation and supervision, and revised the manuscript. AA, EE, NE, EH, ML, FE-G, and EKE participated in the methodology, formal analysis, data curation, and scientific advice. AG, AE, SK, EE, and EKE drafted the manuscript, prepared the manuscript for publication, and revised the manuscript. All authors have read and agreed to the published version of the manuscript.

## Conflict of Interest

The authors declare that the research was conducted in the absence of any commercial or financial relationships that could be construed as a potential conflict of interest.

## Publisher's Note

All claims expressed in this article are solely those of the authors and do not necessarily represent those of their affiliated organizations, or those of the publisher, the editors and the reviewers. Any product that may be evaluated in this article, or claim that may be made by its manufacturer, is not guaranteed or endorsed by the publisher.
